# RecoverEsupport—A Digital Health Intervention for Recovery After Breast Cancer Surgery: Feasibility and Acceptability Outcomes from a Pilot Randomized Controlled Trial

**DOI:** 10.2196/90063

**Published:** 2026-07-09

**Authors:** Emma Sansalone, Erin Forbes, Anna Palazzi-Parsons, Alison Zucca, Mitch J Duncan, Owen James Morris, Stephen Smith, Rebecca Chenery, Helen Moore, Levina Sugono, Priscilla Viana Da Silva, Rebecca Wyse

**Affiliations:** 1 School of Medicine and Public Health, University of Newcastle, 150 University Drive, Callaghan, New South Wales, 2308, Australia, 61 2 4042 0964; 2 Hunter Medical Research Institute, 1 Kookaburra Circuit, New Lambton Heights, New South Wales, Australia, 61240420000; 3 Centre for Active Living and Learning, University of Newcastle, Callaghan, New South Wales, Australia; 4 Calvary Mater Newcastle Hospital, Waratah, New South Wales, Australia

**Keywords:** breast surgery, behavior, digital technology, breast cancer, eHealth, patient-centered care, perioperative care

## Abstract

**Background:**

Optimizing recovery following breast cancer surgery is critical for restoring usual function, minimizing complications, and enabling timely initiation of adjuvant therapies. Enhanced Recovery After Surgery protocols are internationally endorsed recommendations and include patient-led behaviors such as early mobilization, early oral intake of fluids and food, postoperative rehabilitation exercises, and multimodal pain management. However, adherence to these behaviors is often suboptimal, and strategies to support patients are limited. Digital health interventions (DHIs) may offer scalable solutions.

**Objective:**

The aim of the study is to assess the acceptability of the RecoverEsupport Breast DHI, designed to increase adherence to patient-led Enhanced Recovery After Surgery recommendations across the perioperative period for breast cancer surgery, and to assess the feasibility of conducting a randomized controlled trial to evaluate it.

**Methods:**

In this single-site, 2-arm randomized pilot trial, conducted at a major cancer hospital in New South Wales, Australia, between July 2024 and October 2025, participants were consecutively recruited from the surgical list at the study site, supplemented by referrals from surgeons’ private rooms, and included individuals having a mastectomy with or without implant-based reconstruction. Participants were allocated to usual care (control) or usual care plus the RecoverEsupport DHI (intervention). Trial feasibility outcomes included participant recruitment, retention, data completeness, and postoperative safety (adverse events). Intervention acceptability was assessed via the System Usability Scale, participant engagement rates, and willingness to recommend the intervention to others undergoing surgery. Descriptive analyses were conducted, and outcomes were compared to prespecified targets and progression criteria.

**Results:**

In total, 23 participants were recruited (control: n=12, intervention: n=11), which was below the target of 70, while participant retention and data completeness were 100% (23/23), both exceeding the targets. No grade 3+ adverse events occurred; minor grade 2 events occurred in both groups. Acceptability outcomes exceeded targets: usability was high (mean System Usability Scale score 83.2, SD 17.7; target >68), 100% (11/11; target >75%) of participants logged in to the DHI at least once, and 88% (10/11; target >75%) would recommend the program to others undergoing surgery. According to prespecified progression criteria, 3 of 4 feasibility targets were met, indicating that a revised recruitment strategy would be required before proceeding. The restrictive eligibility criteria may have contributed to the lower than expected recruitment rate. All 3 acceptability targets were met.

**Conclusions:**

The RecoverEsupport intervention was acceptable and safe and had high participant engagement. The trial processes were feasible; however, recruitment barriers, including restrictive eligibility criteria, highlight the need for more robust and integrated recruitment strategies to enable progression to a fully powered randomized controlled trial.

## Introduction

Optimizing recovery following surgery is a critical part of the overall treatment pathway, with timely and comprehensive recovery essential to minimize complications and costs, restore function, and allow patients to resume their daily lives [1,2]. In cancer care, optimal recovery from surgery also supports timely initiation of adjuvant therapies such as chemotherapy or radiotherapy, which are often time-sensitive for treatment efficacy [1]. Enhanced Recovery After Surgery (ERAS) protocols are evidence-based, multidisciplinary care pathways recognized internationally as best-practice perioperative care, integrating surgical, anesthetic, nursing, nutritional, and rehabilitative strategies to promote faster, safer surgical recovery [3]. In addition to recommendations to be implemented by clinicians (eg, multimodal analgesia, prophylactic antibiotics, and standardized anesthetic protocols), ERAS protocols also include patient-directed recommendations that require patients to initiate or actively engage in specific behaviors across the perioperative journey, including preoperative, postsurgical, and postdischarge phases. Adherence to these patient-led ERAS behaviors, such as early mobilization, early oral intake of fluid and food, postoperative rehabilitation exercises, and multimodal pain management, has been shown to significantly improve recovery outcomes. For example, adherence to early mobilization recommendations and early resumption of oral feeding is associated with reduced postoperative complication rates in patients undergoing colorectal surgery [4].

ERAS protocols were initially developed for colorectal surgery [5] and have since been refined and expanded to include other surgery types. More recently, ERAS protocols for breast surgery have been published and include a range of patient-led recommendations such as early mobilization, early oral intake of food and fluids, postoperative exercises, and pain management [6]. However, despite the high volume of breast surgeries and substantial complication rates, ERAS protocols remain underused in this surgery type. The majority of the 2.3 million women worldwide diagnosed with breast cancer each year [7] will undergo surgery [8], with around 40% receiving a mastectomy [9]. Around 10% of patients experience postoperative complications such as infection, seroma, or hematoma [8], which can lead to prolonged hospital stays, delayed wound healing, increased pain, and a higher likelihood of readmission. These complications may disrupt planned cancer treatments, requiring chemotherapy or radiation therapy to be delayed [1].

Digital health interventions (DHIs) have been increasingly used in surgical populations to support recovery and adherence to ERAS behaviors. These interventions, including mobile apps, web-based platforms, and wearable devices, have demonstrated improved patient engagement, adherence, and clinical outcomes in colorectal [10-13], hip and knee arthroplasty [14], and other major elective surgery populations [15-17]. However, evidence evaluating perioperative DHIs specifically for women undergoing breast cancer surgery remains limited [18]. Patients undergoing breast surgery represent a distinct surgical population, and their perioperative recovery needs may differ from other surgery groups, highlighting the importance of evaluating DHIs specifically in this context. Limited research has examined patient adherence to ERAS behaviors and interventions to support these behaviors for this patient group. A 2019 systematic review by Offodile et al [19] evaluated the implementation of ERAS protocols in patients undergoing breast reconstruction and reported clinical benefits including reduced hospital length of stay and lower opioid use. A 2023 retrospective observational study [20] examined the implementation of ERAS pathways for mastectomy patients and found similar improvements in perioperative outcomes (eg, shorter length of stay, reduced postoperative pain, and lower complication rates). However, these studies focused on implementing hospital-based interventions to standardize clinical care, rather than developing or testing interventions delivered directly to patients. As a result, there remains a limited understanding of effective strategies to support and increase adherence to patient-led ERAS behaviors in breast surgery.

Evidence from other surgical populations shows that adherence to ERAS behaviors such as early mobilization and early oral intake is often variable and frequently low [21]. Multiple barriers to adherence have been identified. For example, poor communication between health care professionals and patients can lead to misunderstanding of recovery instructions and lower patient adherence [22]. Limited patient-focused education, including insufficient guidance on postoperative exercises, nutrition, and wound care, has also been identified as a key contributor to suboptimal adherence [23]. Finally, lack of continuity of care after discharge, such as inconsistent follow-up or limited access to support services, further reduces patients’ ability to maintain recovery behaviors [24].

DHIs may provide a scalable, cost-effective, and personalized solution to overcome these barriers [25,26]. Evidence indicates that dynamically tailored digital interventions, apps combined with activity trackers, and patient-centered platforms can increase adherence to perioperative recovery behaviors [13,15,18,27]. However, most existing interventions are not behaviorally grounded, target only a subset of ERAS behaviors, and rarely account for the specific needs of patients with breast cancer [11,13,18].

While digital interventions have been developed to support adherence to ERAS behaviors among patients undergoing colorectal [10,12,13,28], hip [29,30], and knee surgery [30,31], to the author’s knowledge, no controlled trials have evaluated such interventions for patients having breast surgery. Furthermore, the existing interventions for other surgery types rarely target the full suite of ERAS behaviors, instead focusing on a small subset (eg, mobilization and oral fluids) [10].

To address these gaps, a behavioral DHI (RecoverEsupport Breast) was developed to support adherence to the patient-led ERAS recommendations across the perioperative pathway in breast cancer surgery. Given the shortage of resources for health care and research, before conducting a large, fully powered randomized controlled trial (RCT) to evaluate intervention effectiveness, it is recommended to first assess whether the trial procedures are feasible and whether the intervention is acceptable to patients currently undergoing surgery [32]. As such, the aim of this pilot RCT was to assess the feasibility of the trial procedures and the acceptability of delivering a DHI to support recovery from breast cancer surgery. It is intended that these findings will inform the design and conduct of a future fully powered RCT.

## Methods

### Study Design

This study was a single-site, 2-arm pilot RCT designed to assess the feasibility of the trial procedures and acceptability of RecoverEsupport, a DHI to increase adherence to the patient-led ERAS behaviors following breast cancer surgery. Participants were randomly allocated in a 1:1 ratio to either usual perioperative care (control group) or usual care plus access to RecoverEsupport (intervention group). The study is reported in accordance with the CONSORT (Consolidated Standards of Reporting Trials) extension for pilot and feasibility trials [33]. The trial was prospectively registered with the Australian New Zealand Clinical Trials Registry (ACTRN12624000417583), and the detailed protocol has been previously published [34]. This paper reports outcomes from analysis of the trial management database, the intervention analytics data, and patient online surveys completed at 1 month after surgery.

### Setting

The trial was conducted in the perioperative clinics and breast surgery unit of a large public cancer hospital in New South Wales, Australia. Recruitment occurred from July 2024 to October 2025.

### Participants

Eligible participants were women aged 18 years or older who were scheduled to undergo a mastectomy for breast cancer (with or without immediate implant-based reconstruction) or a delayed implant-based reconstruction following a previous mastectomy at the study hospital site between July 2024 and October 2025. Participants were required to speak English, have internet access and an email address, and be able to provide informed consent. Patients were excluded if they were undergoing a prophylactic mastectomy and/or autologous reconstruction (eg, deep inferior epigastric perforator, transverse rectus abdominis myocutaneous, or latissimus dorsi flaps), required emergency surgery, were identified by clinical staff as experiencing cognitive impairment that would prevent participation, or if the clinical staff determined that participation would cause them undue distress or anxiety.

### Ethical Considerations

This study has been approved by the Human Research Ethics Committees of the Hunter New England Local Health District (2022/ETH02010), the University of Newcastle (H-2023‐0298), and the Calvary Mater Newcastle (2022/STE03757). Informed consent was obtained from all participants prior to participation in the study. Participant data were deidentified prior to analysis, and all information was stored securely to protect participant privacy and confidentiality. Participants did not receive compensation for participation in this study.

### Recruitment

Patients were identified through perioperative clinics, either in the public hospital or surgeons’ consulting rooms, and eligibility was confirmed by the clinical staff through a standard screener comprising the above criteria. Eligible patients received a study flyer in the clinic prior to being approached by hospital staff either in person or via telephone. Those who expressed interest were emailed a participant information sheet, consent form, and a link to the online baseline survey. Where permission was given, a research assistant (ES) followed up via phone and/or email to answer questions and confirm interest. Ongoing meetings with clinical staff were conducted to identify additional opportunities and strategies for increasing recruitment and resulted in inviting patients from the surgeon’s consulting rooms to ensure patients who do not attend the public clinics were not missed.

### Randomization

Consenting participants were randomized via REDCap (Research Electronic Data Capture; Vanderbilt University) using a concealed, computer-generated sequence in randomly permuted block sizes of 4 to 6 [35]. Participants who completed the baseline survey were randomized immediately after survey completion. If participants had not completed the baseline survey 5 days prior to their date of surgery, randomization was triggered to ensure timely allocation and intervention delivery. Due to the nature of the behavioral intervention, blinding of participants was not possible. Following randomization, participants received an automated study email indicating their allocation. Intervention participants were provided with access details for the RecoverEsupport platform, whereas control participants received an email thanking them for participation and informing them that they would be followed up according to the study protocol. Clinicians and participants were unaware of allocation at the time of enrollment.

### Control Group

All participants received usual perioperative care, which included preoperative consultation with the surgical team and cancer care nurse, standard written postoperative instructions, and community nursing support for patients discharged with surgical drains.

### Intervention Group

**Figure 1.**
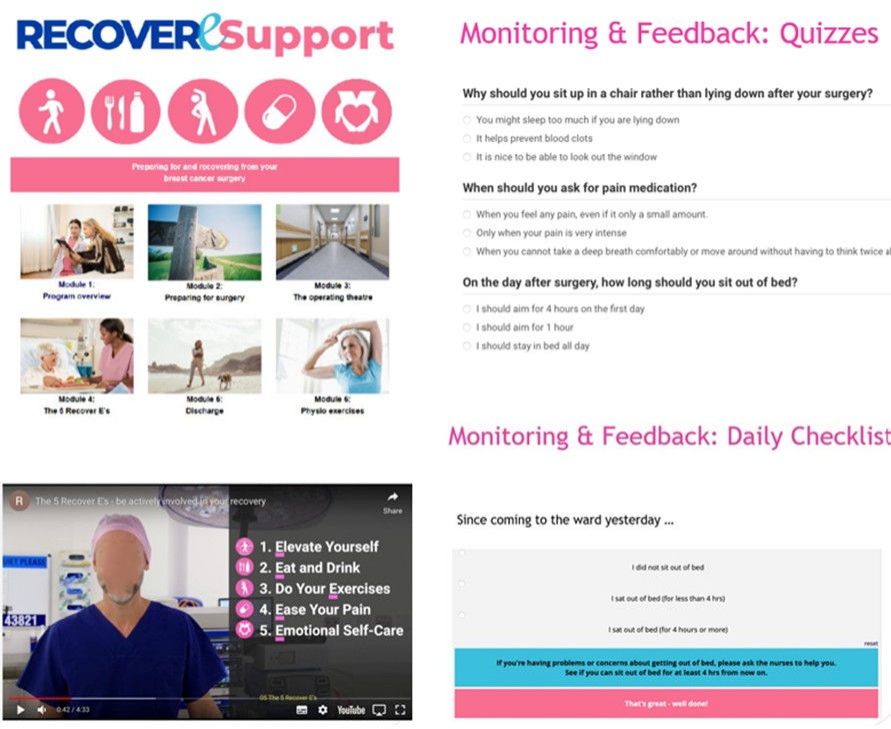
RecoverEsupport digital health intervention used in a single-site pilot randomized controlled trial among women undergoing breast cancer surgery in New South Wales, Australia (July 2024-October 2025). The figure shows the RecoverEsupport online program home screen, clinician-delivered educational video content addressing key recovery behaviors, and the monitoring and feedback component consisting of quizzes and a daily checklist completed by patients during their hospital admission.

In addition to usual care, participants randomized to the intervention group received access to RecoverEsupport. RecoverEsupport is a web-based DHI adapted from an existing tool for colorectal cancer surgery [36], developed using feedback from clinicians, consumers, and patients undergoing breast cancer surgery [37], and informed by the COM-B (Capability, Opportunity, Motivation–Behavior) behavior change framework [38]. The program combined evidence-based behavior change strategies, including provision of education, demonstrations, daily self-monitoring tools, real-time feedback, and automated reminders to support perioperative recovery behaviors such as early mobilization, early food and fluid intake, postoperative rehabilitation exercises, and pain management. Figure 1 shows the home page of the online intervention, including examples of the surgeon’s video, quiz questions, and the daily patient checklist. Participants could access the intervention on demand from presurgery until 3 months postoperatively. Use data (analytics) were automatically collected via REDCap. Participants were encouraged to access the intervention daily during their hospital stay via email reminders. Internet access within some areas of the hospital was sometimes unreliable; therefore, paper checklists were provided as a contingency during hospitalization. These checklists replicated the online versions and allowed participants to tick which ERAS behaviors they had completed in the previous 24 hours and provided brief feedback. Paper-based checklist data were collected by the breast care nurse during the inpatient stay and entered into the standardized form in the REDCap database.

### Primary Outcomes

This paper reports feasibility and acceptability measures collected up to 1 month after surgery using multiple sources, including trial management records, participant-reported online surveys, and intervention analytics data. The primary outcomes focused on feasibility and acceptability and were consistent with established frameworks for pilot and feasibility trials, including the CONSORT extension for pilot and feasibility studies [33] and the conceptual framework proposed by Bowen et al [39]. In line with these frameworks and the published protocol [34], 4 prespecified criteria were evaluated: recruitment, retention, data completeness, and safety or acceptability. Each criterion was assessed using a traffic-light “Stop-Amend-Go” framework [40,41], with targets defined a priori to indicate the extent to which the component should be modified, that is, “Go”—proceed without modification, “Amend”—proceed with minor modifications, or “No-Go”—do not proceed without major modifications. Intervention acceptability was determined using 3 prespecified criteria relating to usability, engagement, and recommendation. All targets were based on either precedent in pilot or feasibility trials or clinical relevance.

### Measures and Data Collection

#### Feasibility

##### Participant Recruitment

This was evaluated based on the number of eligible participants who consented to the trial. A target of 70 participants was deemed sufficient to assess feasibility, aligning with pilot trial recommendations [40]. The nurses conducting the recruitment recorded all patients undergoing breast cancer surgery who were screened for the study in a REDCap “Eligibility” database. They recorded the patients who were invited, eligible, and consented. At the end of the trial, the number of patients who had eligible surgeries was independently verified from medical records as a quality measure. Targets: ≥70 recruited participants (“Go”); 36‐69 participants (“Amend”); and <35 participants (“No-Go”).

##### Participant Retention

This was defined as the proportion of participants remaining in the trial at the 1-month follow-up. Any withdrawals (via email or phone to the study staff or via a request to the nurses) were documented by the study team in the REDCap trial management database. Targets: ≥85% who had not withdrawn at 1 month (“Go”); 51%‐84% (“Amend”); and ≤50% (“No-Go”).

##### Completeness of Data Collection

This was defined as the proportion of participants with available hospital length of stay data extracted from medical records. Length of stay was selected, as this is likely to be the primary outcome for a future fully powered RCT. Following each patient’s surgery, nurses received an automatically generated email prompting them to enter the patient’s discharge date into the REDCap trial management database. Study staff periodically verified completion and sent additional email reminders as needed. Completion rates were assessed against predefined feasibility targets: ≥85% of patients with completed length of stay data (“Go”); 51%‐84% (“Amend”); and ≤50% (“No-Go”).

##### Safety or Adverse Events

Safety was monitored using participant-reported adverse events based on the Common Terminology Criteria for Adverse Events (version 5.0) [42] (Multimedia Appendix 1) because implementation of the program by hospitals and clinicians relies on the intervention being relatively free of adverse effects. Participants were asked in the 1-month survey to report if they had experienced any adverse events relating to falls, muscle pain or discomfort, or anxiety. Target: highest reported adverse events are grade 1 (“Go”); highest reported adverse events are grade 2 (“Amend”); and any reported adverse events of grade 3 or higher (“No-Go”).

### Acceptability (Intervention Participants Only)

#### System Usability

This was assessed via the System Usability Scale (SUS), a valid and reliable 10-item tool for assessing intervention usability [43]. Higher scores represent a more usable intervention, and a score of 68 or above is recommended [43]. As such, the target was set at an average score of 68 or higher. These questions were included in the 1-month online survey.

#### Intervention Engagement

This was measured using the analytics data automatically recorded by REDCap. The target was set at ≥75% of intervention participants logging in at least 1 time to the RecoverEsupport intervention (ie, clicking on the access link).

#### Intervention Recommendation

This was defined as whether or not a participant would recommend the intervention to someone else going through surgery. This question was also included in the 1-month online survey. The target was set at ≥75% of participants, indicating they would recommend the intervention.

### User Experience

Additional data were collected to provide further insight about the participant experience of using RecoverEsupport. These were collected to inform future refinement and implementation of the intervention.

Analytics data were automatically collected via REDCap, including the number of times participants logged in to the website, the number of times they accessed the home page, the number of times they accessed each module, the number of quizzes that were completed, the number of times each video was viewed, and the number of daily checklist entries. For participants with limited internet access on the ward, daily checklist entries could be completed via pen and paper, and this was added to the analytics data in the 1-month survey. Participants were asked a series of study-specific questions to capture their perceptions of the RecoverEsupport program. The questions evaluated aspects such as relevance of content, notifications, checklist utility, preparedness for recovery, and understanding of the information provided.

### Sample Size

The sample size for this pilot study was determined pragmatically. A total of 70 participants (35 per group) was considered sufficient to assess feasibility and acceptability outcomes, in line with previous recommendations for pilot trials [32]. This sample size allows preliminary estimation of recruitment, retention, data completeness, and adverse events, as well as initial indications of acceptability, but the study was not deliberately powered to be able to detect between-group differences or definitive intervention effects.

### Analysis

#### Overview

Baseline characteristics of participants are summarized descriptively. Continuous variables (eg, age) are reported as means and SDs, and categorical variables (eg, sex, gender, marital status, and education level) are reported as counts and percentages. Feasibility and acceptability outcomes were summarized descriptively and compared against the prespecified targets described in the study protocol [34].

#### Overall Recommendation

The decision regarding progression to a fully powered trial was guided by prespecified feasibility and acceptability targets aligned with the Stop-Amend-Go framework. In accordance with recommendations for pilot and feasibility trials, progression was not determined by any single criterion in isolation, but was considered holistically across feasibility domains, including recruitment, retention, data completeness, safety, and acceptability [41]. These domains were interpreted collectively in consultation with the research and clinical team to inform whether the intervention and trial procedures were suitable for progression to a definitive trial with or without modification. Meeting at least 3 of the 4 feasibility domains (ie, “Go” or “Amend”), in the absence of any grade ≥3 adverse events, was used as a structured guide to inform this decision-making process rather than as a strict algorithmic threshold.

## Results

### Overview

**Figure 2.**
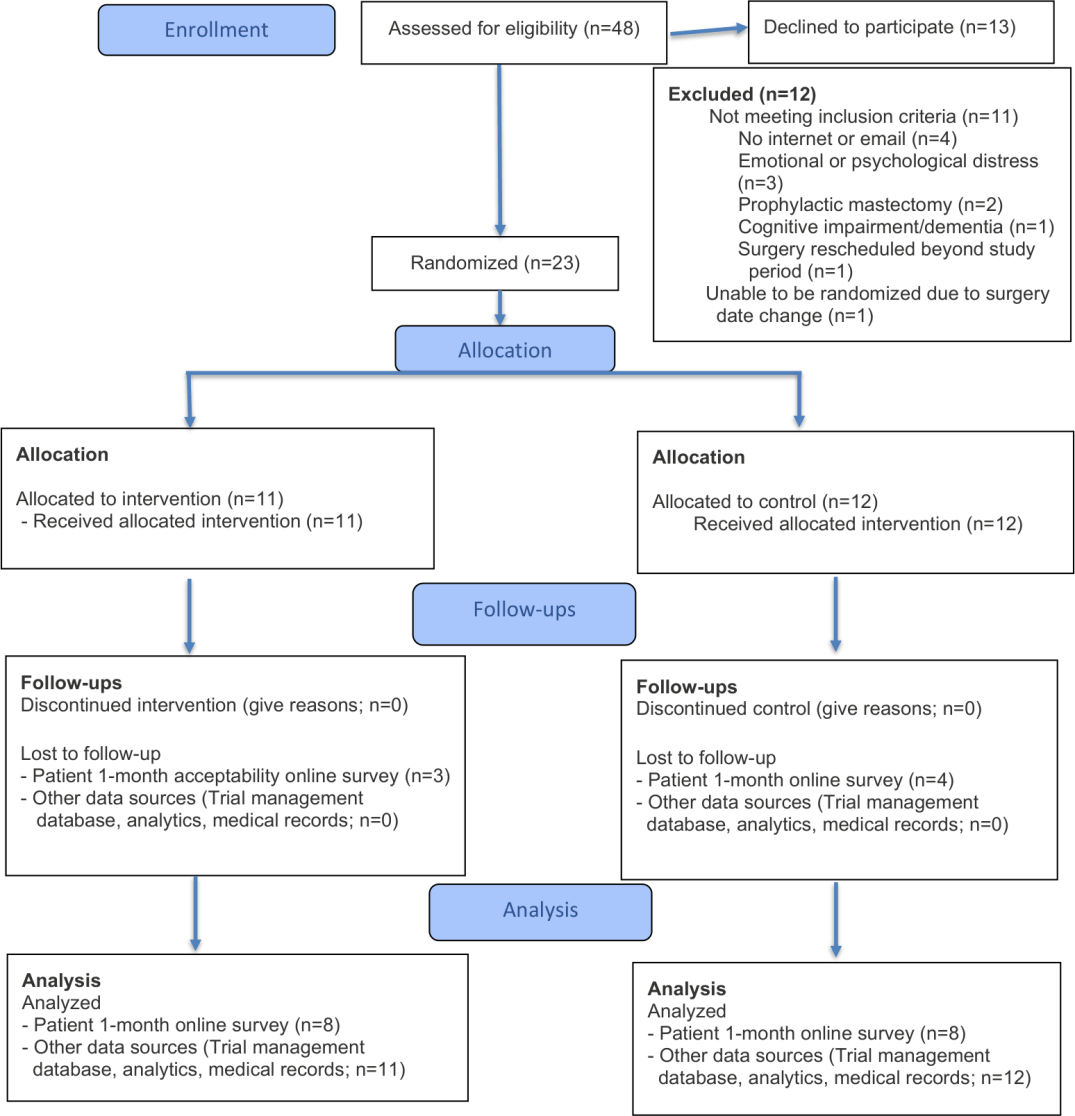
CONSORT flowchart showing participant progression through the trial. This diagram illustrates the flow of participants from initial eligibility assessment through allocation, follow-up, and analysis. A total of 48 participants were assessed for eligibility between July 2024 and October 2025. Of these, 11 were excluded for not meeting the inclusion criteria, 1 participant had their surgery date brought forward before randomization, and 13 declined to participate. In total, 23 participants consented and were randomized to either the intervention (n=11) or the control group (n=12). At 1-month postsurgery follow-up, 3 participants in the intervention group and 4 participants in the control group were lost to follow-up for the online survey. Consequently, 1-month survey data were analyzed for 8 participants in each group. Administrative and medical record data were available for all randomized participants. CONSORT: Consolidated Standards of Reporting Trials.

A total of 48 patients were assessed for eligibility between July 2024 and October 2025. Of these, 36 participants were eligible and invited to participate, and 23 consented to enroll in the study (Figure 2). Recruitment occurred throughout the planned funded study period (July 2024-October 2025). Despite refinement of recruitment strategies in response to a lower than anticipated recruitment rate, the target sample size of 70 was not achieved within this time frame, and recruitment closed as per the funding schedule. Baseline characteristics of the 23 participants are presented in Table 1. The control (n=12) and intervention (n=11) groups were broadly similar across demographic variables. The mean age was 58 (SD 14; range 29‐87) years, with participants in the control group slightly older (61 vs 54 years). All participants were female. Most were born in Australia (21/23, 91%), and less than 5% (1/23) identified as Aboriginal or Torres Strait Islander. Marital status was comparable between groups, with the majority married or living with a partner (15/23, 65%). Educational attainment was similar between groups, with most participants having completed year 10 or trade or vocational training combined (21/23, 91%). Participants resided in major cities (12/23, 52%) or inner regional areas (11/23, 48%). All participants reported using the internet, with most (15/23, 94%) accessing it multiple times per day. Employment status (employed: 10/23, 43%), prior surgical admissions (13/23, 81%), and living arrangements were relatively consistent across groups, with 65% (15/23) living with a partner and 26% (6/23) living alone. With respect to prior cancer treatments, approximately half had not received any previous therapy (12/23, 52%). Prior treatment exposures were generally balanced across groups.

**Table 1.** Baseline demographics and characteristics of participants in the RecoverEsupport pilot randomized controlled trial for patients undergoing breast cancer surgery, New South Wales, Australia (July 2024-October 2025)^a^.

Characteristic	Control (n=12)	Intervention (n=11)	Overall (N=23)
Age (years), mean (SD)	61 (11)	54 (16)	58 (14)
Female (sex), n (%)	12 (100)	11 (100)	23 (100)
Born in Australia, n (%)	12 (100)	9 (82)	21 (91)
Marital status, n (%)			
Divorced or separated	4 (33)	1 (9)	5 (22)
Married or living with partner	7 (58)	8 (73)	15 (65)
Single	0 (0)	1 (9)	1 (4)
Widowed	1 (8)	1 (9)	2 (9)
Highest level of education, n (%)			
Year 10 or school certificate or lower	5 (42)	5 (45)	10 (43)
Trade or vocational training (eg, technical and further education or college)	6 (50)	5 (45)	11 (48)
Bachelor degree	1 (8)	1 (9)	2 (9)
Remoteness, n (%)			
Major cities of Australia	9 (75)	3 (27)	12 (52)
Inner regional Australia	3 (25)	8 (73)	11 (48)
Internet time, n (%)			
Multiple times a day	8 (89)	7 (100)	15 (94)
Once a day	1 (11)	0 (0)	1 (6)
Language other than English, n (%)	0 (0)	0 (0)	0 (0)
Currently employed, n (%)	5 (42)	5 (45)	10 (43)
Covered by private health insurance, n (%)	0 (0)	0 (0)	0 (0)
Previously admitted to hospital for surgery, n (%)	8 (89)	5 (71)	13 (81)
Lives with, n (%)			
Spouse or partner	7 (58)	8 (73)	15 (65)
Children	3 (25)	4 (36)	7 (30)
Other family	1 (8)	1 (9)	2 (9)
Lives alone	4 (33)	2 (18)	6 (26)
Previous treatments, n (%)			
None	7 (58)	4 (45)	12 (52)
Chemotherapy only	2 (17)	0 (0)	2 (89)
Chemotherapy+immunotherapy	1 (8)	2 (18)	3 (13)
Unknown or not reported	2 (17)	3 (27)	5 (22)

a The control group received usual perioperative care (n=12), and the intervention group received usual care plus access to RecoverEsupport (n=11).

### Feasibility and Acceptability Outcomes

Feasibility and acceptability outcomes for the pilot RCT are presented in Table 2. Recruitment did not meet the target, with 23 participants enrolled compared with the target of 70, resulting in a “No-Go” progression status. Trial retention at 1-month follow-up was 100% (no active withdrawals from the study), exceeding the ≥85% target and meeting the “Go” progression status. Completeness of hospital length of stay data collection from hospital medical records was 100%, meeting the predefined target and the “Go” progression status.

**Table 2.** Feasibility and acceptability progression criteria outcomes in the RecoverEsupport pilot randomized controlled trial with patients undergoing breast cancer surgery at 1 Australian hospital (July 2024-October 2025).

Domain and outcome measure	Target	Observed result	Progression status^a^
Feasibility			
Participant recruitment	≥70 participants consented	23	No-Go^b^
Retention at 1-month follow-up	≥85% not actively withdrawn	100%	Go^c^
Collection of length of stay data	≥85% have complete data	100%	Go^c^
Adverse events at 1 month in the intervention group for falls, muscle pain or discomfort (soreness), and anxiety	Only grade 1 events)	Falls: No adverse events reported Anxiety: No adverse events reported Muscle pain or discomfort (muscle soreness): Grade distribution reported across all participants: Intervention group: grade 0: 56%, grade 1: 33%, grade 2: 11%, grade 3: 0% Control group: grade 0: 89%, grade 1: 0%, grade 2‐: 11%, grade 3: ‐0%	Amend^d^
Acceptability			
System Usability Scale	Mean score >68	Mean score=83.2	Go^c^
Platform engagement	≥75% log in once or more	100% participants	Go^c^
Would recommend RecoverEsupport to others	≥75% would recommend	88% would recommend	Go^c^

a Progression status was determined using prespecified Stop-Amend-Go criteria applied across feasibility domains and safety outcomes.

b No-Go criterion met due to recruitment below predefined threshold (<70 participants).

c Go criterion met based on predefined Stop-Amend-Go thresholds.

d Amend criterion was met due to the presence of grade 2 adverse events; no grade ≥3 adverse events were reported.

At 1-month follow-up, no participants in either group reported any adverse events related to falls or anxiety. Among intervention participants (n=11), 56% (6/11) reported no adverse events related to muscle soreness, 33% (4/11) reported grade 1 mild muscle pain, and 11% (1/11) reported grade 2 moderate muscle pain (Table 2). In the control group, 89% (11/12) reported no adverse events related to muscle soreness, no grade 1 events were reported, and 11% (1/12) reported grade 2 moderate muscle pain. No grade 3 or higher events were reported in either group. Although overall adverse event rates were low, the presence of grade 2 events (even though evenly distributed across both groups) meant that the outcome did not fully meet the predefined feasibility “Go” criterion, which allowed only grade 0‐1 events. Consistent with the protocol, the occurrence of grade 2 events resulted in an “Amend” progression status, indicating that minor modifications to the intervention or safety-monitoring procedures may be warranted before progressing to a definitive trial. All acceptability outcomes met or exceeded targets. The mean SUS score was 83.2 (SD 17.4; range 50‐100), well above the target of 68. Platform engagement was high, with 100% (11/11) of participants logging in at least once (see Table 2 and Figure 2 for complete engagement data), and 88% of participants reporting that they would recommend the program to others, both exceeding the predefined targets.

Overall, the pilot study demonstrated full participant retention and complete data collection and met all intervention acceptability targets. Recruitment fell substantially below the predefined target (23/70 participants), corresponding to a “No-Go” outcome, indicating that substantial modifications to the recruitment strategy would be required before progression to a fully powered RCT. Grade 2 adverse events of moderate muscle pain were observed in a minority of participants.

### Supplementary Data: User Experience With the RecoverEsupport Intervention

All participants in the intervention group had available engagement data from REDCap. Overall, 100% of participants logged in at least once (ie, each participant clicked the link that was sent to them via email and accessed the platform). This measure captures access to the intervention at any point, but does not reflect whether or not they accessed or completed modules, or the extent of active engagement with content. Among the 7 participants who accessed the modules, they accessed each module an average of 1.4 times. The proportion of participants who completed each of the 6 modules ranged from 45% to 64%. In total, 3 (27%) participants completed all 6 modules, while 4 (36%) participants did not engage with any module content despite accessing the home page. With respect to goal setting, 5 of the 11 (45%) participants accessed the goal-setting module, and 3 of 11 entered responses into the module, but only 1 participant set a goal that would be considered a SMART (Specific, Measurable, Achievable, Relevant, and Time-bound) goal. Use of additional program features varied. All participants (11/11, 100%) completed at least 1 daily checklist entry, and 8 (73%) participants completed daily checklists every day during their hospital admission. In total, 2 participants completed checklist entries via pen and paper due to internet access issues on the hospital ward. Most participants (7/11, 64%) answered at least 1 quiz question, while 3 (27%) participants completed all available questions. Nearly half of the participants (5/11, 45%) watched at least 1 video (ie, had the video webpage open for at least 80% of the duration of the video), but only 1 (9%) participant watched all videos.

Participant perceptions of the intervention were assessed at 1 month after surgery. Among participants who completed the acceptability items in the 1-month follow-up survey (n=8), the intervention was rated as relevant by 100% (8/8) of participants, 75% (6/8) reported that the alerts and daily checklists were acceptable, 88% reported understanding the content, and 100% (8/8) reported feeling adequately prepared for postoperative recovery following use of the program (Multimedia Appendix 2).

## Discussion

### Principal Findings

This pilot study aimed to evaluate the feasibility of trial procedures and the acceptability of the RecoverEsupport DHI for women undergoing breast cancer surgery. Overall, the trial procedures demonstrated strong feasibility in several domains: participant retention was high (100%) with no active withdrawals, and completeness of hospital length of stay data was excellent (100%) based on extraction from hospital medical records. However, recruitment did not meet the predefined target (23 participants enrolled vs ≥70 required), resulting in a “No-Go” outcome for this domain. Furthermore, this intervention was considered acceptable, with SUS scores, intervention engagement, and participant willingness to recommend the program exceeding predefined targets. These findings suggest that while the trial procedures and intervention delivery are generally feasible, recruitment processes will require modification in a future definitive trial.

### Comparison to Prior Work

Several factors contributed to the failure to meet the recruitment target. The target sample size was based on the patient volume through the hospital in the 5-year period prior to the study commencing. However, surgical and reconstruction practices are evolving in Australia and internationally, with the relative number of autologous surgeries increasing and the proportion of mastectomies decreasing in favor of breast-conserving surgery (eg, lumpectomies) [44]. As such, the historical patient volume data may have overestimated the number of eligible patients within the trial period. This explanation is supported by a medical records audit over the trial period to identify potential eligible patients who were not invited, identifying only 2 potentially eligible patients who were missed. This suggests that the recruitment approach was effective in identifying and contacting potential participants, but that the exclusion criteria may have been too restrictive. In addition to these surgical trends, the trial faced operational recruitment challenges. For example, last-minute changes to scheduled surgery dates sometimes resulted in patients becoming ineligible for the study. For example, one participant had their surgery date brought forward and could not be randomized with sufficient time prior to surgery. Difficulty in meeting recruitment targets is common to trials recruiting patients with cancer during active treatment, including during the perioperative period [45,46]. Future trials should address such recruitment challenges through less restrictive eligibility criteria, the involvement of multiple recruitment sites, and the integration of study procedures into routine clinical workflows. Embedding recruitment strategies within the electronic medical records and routine care (eg, automated eligibility screening and/or reminders) has been shown to streamline identification of participants, reduce screening burden, and improve recruitment efficiency [47].

### Safety Outcomes

Safety outcomes were generally acceptable, with no adverse events reported for falls or anxiety. Grade 2 muscle soreness events were reported evenly in both the intervention and control groups, suggesting that they were more likely attributable to routine postoperative recovery rather than to the intervention itself. Consistent with this, clinician advice suggests that postoperative muscle soreness, which is common after breast surgery and typically managed with simple analgesia without limiting activities of daily living, should not automatically be classified as an adverse event that would warrant stopping the intervention. Posttrial discussion with the clinicians suggested that, for future trials, the threshold for an adverse event should be increased to events of grade 3 or higher. The low rates of muscle soreness and the absence of falls or anxiety suggest that RecoverEsupport is safe for future implementation. The reporting of adverse events is important information that hospitals and health systems consider in their decision to adopt novel interventions. Despite this, to date, studies of DHIs in surgical settings have not routinely or explicitly reported adverse events resulting from intervention use, with studies more commonly reporting readmissions or complications from surgery [10,12,13,30,31].

### Intervention Engagement

The difference in engagement rates between intervention components was notable. Of the 11 participants allocated to the intervention group, 7 accessed the online modules. Among those who accessed modules, module completion (defined as viewing ≥50% of module video content) ranged from 45% to 64%, whereas all participants (100%) completed at least 1 daily checklist entry, with 73% completing checklists every day during their hospital admission. There are multiple explanations for differences in engagement rates between these different intervention components. First, the nature of the component may play a role. Structured and frequent self-monitoring, such as checklists, often show high completion because these tasks are quick, simple, and easy to integrate into daily routines [48,49]. In contrast, the modules, comprised of written information, videos, and quizzes, took longer to complete (an estimated 5‐10 minutes) and were more cognitively demanding. Second, the timing of delivery of each intervention component may have influenced engagement. Although all surgeries were planned, and participants had access to the modules prior to their hospital admission—a time when patients typically seek information and support—engagement within the modules was only moderate. This aligns with studies of other digital interventions for surgical patients [50], highlighting the challenges of engaging with patients during the perioperative period, where there are often competing demands on patients’ attention and capacity. In contrast, the checklists were delivered postoperatively while the patient was still in the hospital, at a time when patients are essentially a more captive audience with fewer demands on their time. Furthermore, perceived clinician involvement may have also played a role. Patient responses to each checklist triggered automatic email alerts to nursing staff. Participants were informed that their responses could be shared with their treating team. This may have contributed to the high engagement with the checklists, consistent with the supportive accountability model, which proposes that adherence to digital interventions increases when users expect their behavior to be monitored by a trusted clinician [51].

The engagement data suggest that future trials could consider refinements to the RecoverEsupport intervention to improve engagement by prioritizing low-burden, structured tasks such as daily checklists that support self-monitoring of patient-led ERAS behaviors. Module content may benefit from simplification, using shorter, more digestible segments to minimize cognitive burden and fatigue, or from tailoring based on engagement. For example, consistent with the emerging field of adaptive interventions, if a participant has not accessed a module prior to surgery, the system could deliver a simplified “essential actions only” summary or push a single, high-priority prompt (eg, a 30-second reminder about the recommended activity for that stage of recovery). Combining educational digital content (eg, videos or informational modules) with interactive self-management tools (eg, checklists and mobilization prompts) may further enhance adherence and promote recovery behaviors as demonstrated in prior DHIs and ERAS-related interventions [13,25]. Intervention refinement could also be informed by collecting qualitative feedback from trial participants. Although the initial development of RecoverEsupport was informed by qualitative interviews with former patients undergoing breast surgery (ie, women having surgery within the past 6‐18 months) [37], it is possible that the experiences of current and former patients differ.

### Strengths and Limitations

The results should be considered with respect to the limitations of the trial. The small sample size and single-site design mean that the extent to which these results generalize to different contexts is unclear. Furthermore, participants were excluded if they were identified as experiencing cognitive or emotional impairment or undue distress, which may limit the generalizability of these findings to more vulnerable patients. Assessment of cognitive or emotional impairment was based on clinician judgment and may therefore be subject to variability, with the potential for both under- and overidentification of individuals who could have participated. However, such judgments are routinely required in clinical practice, and to support consistency, the nurses were provided with guidance, descriptions, and examples within the screening tool to standardize decision-making across clinicians. A further limitation is the absence of qualitative feedback from trial participants exploring their experiences of the intervention. Intervention refinement would benefit from additional qualitative evaluation to better understand intervention acceptability, the usefulness of specific components, and mechanisms of engagement.

### Future Directions

Future research should address the recruitment challenges identified in this pilot study through less restrictive eligibility criteria, recruitment across multiple sites, and integration of study procedures into routine clinical workflows. Embedding recruitment strategies within electronic medical records and routine care may improve recruitment efficiency and reduce screening burden.

Future iterations of RecoverEsupport may also benefit from intervention refinements to enhance engagement. These may include prioritizing low-burden, structured self-monitoring tasks such as daily checklists, simplifying educational content into shorter and more digestible segments, and incorporating adaptive intervention strategies based on participant engagement. In addition, qualitative evaluation with trial participants would provide valuable insights into intervention acceptability, the usefulness of specific components, and mechanisms influencing engagement.

### Conclusions

Pilot studies play a critical role in intervention research, providing necessary insights into key processes that will ultimately determine the success of a fully powered trial, such as recruitment and retention processes, intervention engagement, and intervention acceptability to participants.

This pilot RCT demonstrates that RecoverEsupport is a safe, acceptable, and engaging DHI to support recovery following breast cancer surgery. Although recruitment did not reach the target sample size, participant retention and data completeness were high, and adverse events were observed across both intervention and control groups, with no safety concerns specifically attributable to the intervention. Overall, these findings provide guidance for refining digital perioperative interventions and conducting future RCTs, supporting scalable implementation to improve recovery outcomes for patients undergoing breast cancer surgery.

## Supplementary material

10.2196/90063 Multimedia Appendix 1 Participant-reported adverse event questions and response options.

10.2196/90063 Multimedia Appendix 2 User experience survey items, response options, and participant responses at 1 month after surgery.

10.2196/90063 Checklist 1 CONSORT checklist.

## Abbreviations

CONSORT: Consolidated Standards of Reporting Trials
DHI: digital health intervention
ERAS: Enhanced Recovery After Surgery
RCT: randomized controlled trial
REDCap: Research Electronic Data Capture
SUS: System Usability Scale
