# Supplementary material for: RecoverEsupport—A Digital Health Intervention for Recovery After Breast Cancer Surgery: Feasibility and Acceptability Outcomes from a Pilot Randomized Controlled Trial
Source: JMIR Form Res. 2026 Jul 9;10:e90063. doi: 10.2196/90063 (PMC13349321; doi:10.2196/90063)

The following questions assessed participant-reported adverse events adapted from the Common Terminology Criteria for Adverse Events (CTCAE).


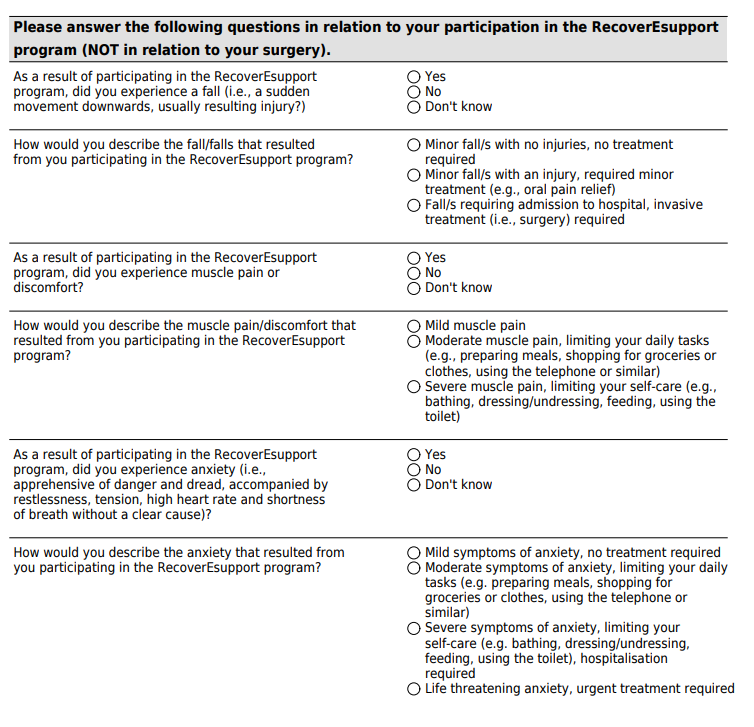

Supplement: Multimedia Appendix 1 [file formative-v10-e90063-s001.docx]
