# Supplementary material for: RecoverEsupport—A Digital Health Intervention for Recovery After Breast Cancer Surgery: Feasibility and Acceptability Outcomes from a Pilot Randomized Controlled Trial
Source: JMIR Form Res. 2026 Jul 9;10:e90063. doi: 10.2196/90063 (PMC13349321; doi:10.2196/90063)
Supplement: Multimedia Appendix 2 [file formative-v10-e90063-s002.docx]

| **Domain** | **Survey item** | **Response options** | **Participant response** |
| --- | --- | --- | --- |
| Intervention relevance | Overall, how relevant to you was the information in the RecoverEsupport program? | Very relevant, Relevant, Irrelevant, Very irrelevant, Unsure | 100% (8/8) reported the program was relevant or very relevant |
| Acceptability of reminder alerts | Receiving the alerts was acceptable | Strongly disagree, Disagree, Neither agree nor disagree, Agree, Strongly agree | 75% (6/8) agreed or strongly agreed |
| Acceptability of reminder alert frequency | The frequency of alerts was acceptable | Strongly disagree, Disagree, Neither agree nor disagree, Agree, Strongly agree | 75% (6/8) agreed or strongly agreed |
| Acceptability of reminder alert content | The content of the alerts was acceptable | Strongly disagree, Disagree, Neither agree nor disagree, Agree, Strongly agree | 75% (6/8) agreed or strongly agreed |
| Acceptability of daily checklists | Completing checklist entries in hospital was acceptable | Strongly disagree, Disagree, Neither agree nor disagree, Agree, Strongly agree | 75% (6/8) agreed or strongly agreed |
| Understanding of intervention content | I understood what to expect after discharge | Strongly disagree, Disagree, Neither agree nor disagree, Agree, Strongly agree | 88% (7/8) agreed or strongly agreed |
| Preparedness for postoperative recovery | I felt adequately prepared for discharge following my surgery | Strongly disagree, Disagree, Neither agree nor disagree, Agree, Strongly agree | 100% (8/8) agreed or strongly agreed |

Percentages represent participants reporting positive responses. For intervention relevance, positive responses were "Relevant" or "Very relevant". For Likert-scale items, positive responses were "Agree" or "Strongly agree".
